# The Memory System Engaged During Acquisition Determines the Effectiveness of Different Extinction Protocols

**DOI:** 10.3389/fnbeh.2015.00314

**Published:** 2015-11-24

**Authors:** Jarid Goodman, Mark G. Packard

**Affiliations:** Department of Psychology, Texas A&M Institute for Neuroscience, Texas A&M UniversityCollege Station, TX, USA

**Keywords:** extinction, memory, hippocampus, striatum, memory systems

## Abstract

Previous research indicates that extinction of rodent maze behavior may occur without explicit performance of the previously acquired response. In latent extinction, confining an animal to a previously rewarded goal location without reinforcement is typically sufficient to produce extinction of maze learning. However, previous studies have not determined whether latent extinction may be successfully employed to extinguish all types of memory acquired in the maze, or whether only specific types of memory may be vulnerable to latent extinction. The present study examined whether latent extinction may be effective across two plus-maze tasks that depend on anatomically distinct neural systems. Adult male Long-Evans rats were trained in a hippocampus-dependent place learning task (Experiment 1), in which animals were trained to approach a consistent spatial location for food reward. A separate group of rats were trained in a dorsolateral striatum-dependent response learning task (Experiment 2), in which animals were trained to make a consistent egocentric body-turn response for food reward. Following training, animals received response extinction or latent extinction. For response extinction, animals were given the opportunity to execute the original running approach response toward the empty food cup. For latent extinction, animals were confined to the original goal locations with the empty food cup, thus preventing them from making the original running approach response. Results indicate that, relative to no extinction, latent extinction was effective at extinguishing memory in the place learning task, but remained ineffective in the response learning task. In contrast, typical response extinction remained very effective at extinguishing memory in both place and response learning tasks. The present findings confirm that extinction of maze learning may occur with or without overt performance of the previously acquired response, but that the effectiveness of latent extinction may depend on the type of memory being extinguished. The findings suggest that behavioral treatments modeled after response extinction protocols may be especially useful in alleviating human psychopathologies involving striatum-dependent memory processes (e.g., drug addiction and relapse).

## Introduction

Mammalian memory is not a unitary phenomenon, but rather it transpires through distinct systems. These “memory systems” differ in terms of not only the type(s) of memory they mediate, but also the brain regions that subserve them. Although a variety of memory systems have been dissociated in the mammalian brain (Squire, 2004; White et al., 2013), significant attention has been devoted to anatomical dissociations between a spatial/cognitive memory system mediated by the hippocampus and a stimulus-response (S-R)/habit system mediated by the dorsolateral striatum (DLS; Packard et al., 1989; Packard and McGaugh, 1992, 1996; McDonald and White, 1993; Packard and Teather, 1997; Chang and Gold, 2003; Iaria et al., 2003; Compton, 2004).

Research from our laboratory indicates that multiple memory systems may not only be implicated in the initial acquisition of a task, but also in its extinction (Gabriele and Packard, 2006). Extinction constitutes a new, dissociable type of learning that occurs when a subject is placed in the original learning situation but with the reinforcer—or the stimulus event that motivated initial learning—removed. Extinction is deemed to have occurred when the behavioral response or responses that indicated initial learning decrease. Learned behavior in the straight alley maze, a maze in which rats learn to traverse a runway for food reward located at the opposite end of the maze, may be extinguished using two distinct protocols. In a typical “response extinction” protocol, rats are placed in the same starting position as during training, but with the food reward at the opposite end of the maze removed. Thus, during response extinction trials, animals can execute the running approach response, only now this response leads to an empty food well. In “latent extinction,” rats are confined to the original goal location with the empty food well. Thus, during latent extinction, animals cannot execute the running approach response. Historically the effectiveness of latent extinction figured prominently in learning theory, because it demonstrated that—in contrast to the Hullian S-R view of extinction (Hull, 1943, 1952)—a subject does not need to make the previously acquired response for extinction to occur (Seward and Levy, 1949; Deese, 1951; Moltz, 1955; Denny and Ratner, 1959; Dyal, 1962; Clifford, 1964).

Although the behavior of the rat is ostensibly similar following both extinction protocols, investigators have suggested that response and latent extinction might be achieved through distinct learning mechanisms. The effectiveness of typical response extinction is easily explained through classical S-R models of extinction learning, whereas latent extinction has summoned heated debates between proponents of expectancy theory and proponents of a neo-Hullian view involving the fractional anticipatory approach response (Moltz, 1957; Deese and Hulse, 1967). Although the precise mechanisms underlying latent extinction have yet to be completely elucidated, evidence from our laboratory indicates that latent extinction indeed depends on a dissociable neural system. In the straight-alley maze inactivation of the hippocampus, but not the DLS, impairs latent extinction (Gabriele and Packard, 2006; Gabriele, 2008). In contrast, inactivation of the DLS, but not the hippocampus, impairs response extinction (Gabriele and Packard, 2006; Gabriele, 2008). A corollary to the contention that these extinction protocols depend on operatively and anatomically distinct learning systems is that response and latent extinction may not be equally effective across all learning situations. For instance, if a critical feature needed for latent extinction mechanisms to occur is absent from the learning situation, then it is reasonable to hypothesize that latent extinction would not be effective, whereas response extinction could still work.

One potential limitation to examining learning and memory mechanisms using the straight-alley maze is that we do not know what type of memory is being encoded during initial task acquisition. Initial learning in the straight alley maze may involve acquisition of at least two distinct types of memory: (1) a habit-like running approach response to the opposite end of the maze and/or (2) the spatial location of the food reward, which in turn triggers a goal-directed running approach to the rewarded location at the opposite end of the maze. Consequently, when using the straight alley maze to examine extinction mechanisms, the type of memory being extinguished remains unknown. Moreover, studies using the straight alley maze cannot determine whether latent extinction is effective at extinguishing all types of memory or whether latent extinction may only be effective for certain types of memory. Considering that latent extinction may partially operate by producing a new inhibitory spatial memory (see Gabriele and Packard, 2006), it is possible that latent extinction may only be effective in tasks whereby the spatial location of the goal is an integral part of the to-be-extinguished memory, such as in spatial memory tasks. In contrast, latent extinction may not be effective in tasks whereby the spatial location of the goal is irrelevant, such as in S-R/habit memory tasks.

To examine whether only certain types of memory may be vulnerable to latent extinction, the present study utilized two distinct versions of the plus-maze. In a “place learning” version of the plus-maze dependent on the hippocampus (Schroeder et al., 2002; Compton, 2004), rats were reinforced to approach a consistent spatial location. In a “response learning” version of plus-maze dependent on the DLS (Chang and Gold, 2004; Palencia and Ragozzino, 2005; Asem and Holland, 2015), rats were reinforced to make a consistent egocentric body-turn at the maze choice point. Thus, these place and response tasks tap into dissociable neural systems, a hippocampus-dependent spatial/cognitive memory system and a DLS-dependent S-R/habit memory system, respectively. Following initial learning in these tasks, animals were given response extinction, latent extinction, or no extinction. It was hypothesized that latent extinction would be selectively effective at extinguishing memory in the place learning task, but not the response learning task. Moreover, we hypothesized that typical response extinction would be effective at extinguishing memory in both place and response learning tasks.

## Materials and Methods

### Subjects

The subjects were 46 male Long-Evans rats approximately 90 days old and weighing 375–425 g upon arrival. Animals were subsequently food-restricted and maintained at 85% of the their ad lib weight throughout all behavioral procedures. Water was provided ad libitum. Animals were housed individually in a temperature-controlled vivarium with a 12 h light-dark cycle (lights on at 7 AM), and all behavioral procedures were conducted during the light phase of this cycle. Age, weight, and housing conditions did not differ between animals in Experiments 1 and 2. Animal use in this study was carried out in accordance with the ethical guidelines of the Institutional Animal Care and Use Committee (IACUC) at Texas A&M University. The protocol was approved by IACUC.

### Apparatus

An eight arm radial maze was modified by removing four of the original arms to create a plus-maze configuration consisting of north, south, east, and west arms. The arms of the cross maze measured 60 × 9 cm, and the center platform of the maze connecting the four arms measured 40 cm in diameter. At the end of each arm was a recessed food well. A clear Plexiglas cross-shaped structure was placed in the center of the cross maze, serving as the intersection of the four arms. A separate Plexiglas divider was used to block off the arm opposite to the start arm for each trial, creating a T-maze configuration that could be adjusted between trials. The maze was situated in a room with multiple extra maze cues, including posters, a door, a cabinet, and a table.

### Behavioral Procedures

#### Maze Habituation

Before maze training, animals in Experiments 1 and 2 were given 2 days of habituation to the maze. For each day of habituation, a rat was placed on the maze apparatus (from the north arm on day 1 and from the south arm on day 2) and was given 5 min to explore the maze. No food was located on the maze at this time. Immediately after the 5 min, each rat was removed from the maze and placed in a holding container with three Froot Loops cereal pieces (Kellog’s). Rats were monitored to confirm consumption of the Froot Loops.

#### Maze Training

Maze training began 24 h following the last day of habituation and lasted 8 days. For the first 2 days of training, animals were given six trials per day, and for the remainder of training animals were given 15 trials per day. The maze was rotated 90° after every two trials to discourage the use of intramaze cues. A wide-angle digital camera was fixed over the maze and attached to a computer monitor (only visible to the experimenter) allowing for a clear aerial view of arm entries, and a stopwatch was used to record latencies during task performance.

In Experiment 1, animals (*N* = 21) received training for 8 days in a place learning version of the plus-maze task whereby animals were reinforced to approach a consistent spatial location. At the start of each training trial, the animal was placed on the north or south arm facing the outside of the maze (the start arm sequence was counterbalanced across training), and the food reward (1/2 Froot Loop) was always located in the recessed food well of the east arm. This place learning protocol presumably compelled rats to acquire a cognitive map of the learning environment that enabled them to guide behavior from different starting positions to the correct spatial location. Extensive evidence indicates that spatial learning in the plus-maze critically involves hippocampal function (Packard and McGaugh, 1996; Packard, 1999; Schroeder et al., 2002; Colombo et al., 2003; Compton, 2004; Jacobson et al., 2012).

In Experiment 2, animals (*N* = 25) received training in a response learning version of the plus-maze task whereby animals were reinforced to make a consistent egocentric body-turn response at the maze choice point (Leong et al., 2012, 2015; Goodman and Packard, 2014; Wingard et al., 2015). Animals were released from north and south starting positions (counterbalanced) throughout training. When animals began in the north arm, the food reward (1/2 Froot Loop) was located in the recessed food well of the east arm. When animals began in the south arm, the food reward was located in the west arm. Thus, regardless of the starting position, animals were reinforced to make a left body-turn response at the choice point to receive food reward. Learning in this task constitutes an exemplar of egocentric/S-R learning mediated by the DLS (Packard and McGaugh, 1996; Chang and Gold, 2004; Palencia and Ragozzino, 2005; Asem and Holland, 2015; for reviews, see Packard, 2009; Goodman and Packard, in press).

For each training trial in Experiments 1 and 2, if the animal made an initial full-body entry into the correct arm (i.e., the arm containing the food), the trial was scored as correct. If the animal made an initial full body entry into the incorrect arm, the trial was scored as incorrect. A trial ended once the animal found the food or after 120 s had elapsed. When finding the food, the animal was allowed to finish eating before being removed from the maze and placed in an opaque holding container for a 30 s intertrial interval (ITI). The percentage of correct trials and the latency to reach the correct food well were used as measures of acquisition.

#### Extinction

Extinction was conducted 24 h after the last day of maze training and lasted 3 days. No food was located in the maze throughout extinction training. The maze was rotated 90° after every two trials to prevent the use of intramaze cues.

In Experiment 1, rats that were previously given place learning were subsequently assigned to response extinction (*n* = 7), latent extinction (*n* = 7), or “no extinction” control (*n* = 7) groups. Groups were matched on average latency and percent correct responses during the last 3 days of acquisition. Response extinction was conducted over 3 days (10 trials per day). For each trial of response extinction, animals were started from the north or south arm and were given the opportunity to run to the previously correct food well. An animal was removed from the maze after reaching the previously correct food well or after 120 s had elapsed. For each trial, if the animal made an initial full-body entry into the previously correct arm and ran directly to the food well, the trial was identified as “perseverative.” A trial was not considered perseverative if the animal at any point made an entry into the incorrect arm or failed to enter either the correct or incorrect arm within 120 s. After each trial the animal was removed from the maze and placed in an opaque holding container for a 30 s ITI. The behavioral procedure for latent extinction was adapted from previous work from our laboratory indicating the effectiveness of latent extinction in the straight alley maze (Gabriele and Packard, 2006, 2007; Gabriele et al., 2009). For each trial of latent extinction, an animal was confined to the previously correct goal arm (i.e., the east arm for the place learning task) for 60 s using a Plexiglas shield secured 20 cm from the end of the maze arm. After each trial, the animal was placed in an opaque holding container for a 30 s ITI. For the “no extinction” control group, animals were not placed in the maze for the 3 extinction days, but rather remained in their holding containers for the duration of an extinction session, i.e., while animals in the latent and response extinction groups were receiving extinction training.

In Experiment 2, animals that previously received response learning were subsequently assigned to response extinction (*n* = 6), limited latent extinction (*n* = 6), extended latent extinction (*n* = 6), or “no extinction” control (*n* = 7) groups. Groups were matched on average latency and percent correct responses during the last 3 days of acquisition. The behavioral procedures for response extinction and no extinction control groups were identical to that described for Experiment 1. For limited and extended latent extinction (conducted over 3 days), animals were confined to the east or west goal arm for 60 s for each trial with the sequence of goal arm confinements mimicking the counterbalanced sequence of food locations throughout initial response learning. For each day of limited latent extinction, animals received 10 trials (five trials on each arm). The parameters for limited latent extinction were chosen based on previous evidence indicating that 10 latent extinction trials per day produced extinction in the straight alley (Gabriele and Packard, 2006). However, given that latent extinction trials had to be divided between east and west goal arms, this only permitted five trials on each arm per day. In order to allow for 10 trials on each arm, an additional group was given extended latent extinction, in which animals received 20 trials (10 trials on each arm) per day.

#### Extinction Probes

Twenty four hours following the last day of extinction, all animals in Experiments 1 and 2 were given four probe trials. No food was located in the maze for the extinction probe trials. For each probe trial, an animal was released from the north or south arm (start arm sequence: SNNS), and after reaching the previously correct food well or after 120 s had elapsed, animals were removed from the maze and placed in an opaque holding container for a 30 s ITI. The maze was rotated 90° after every two trials. Latency to reach the previously correct food well and the number of perseverative trials (see above) were recorded and used as measures of extinction. The experimenter conducting the probe trials and scoring the animals was blind to the experimental conditions.

## Results

### Experiment 1

#### Initial Acquisition

**Figure 1:**
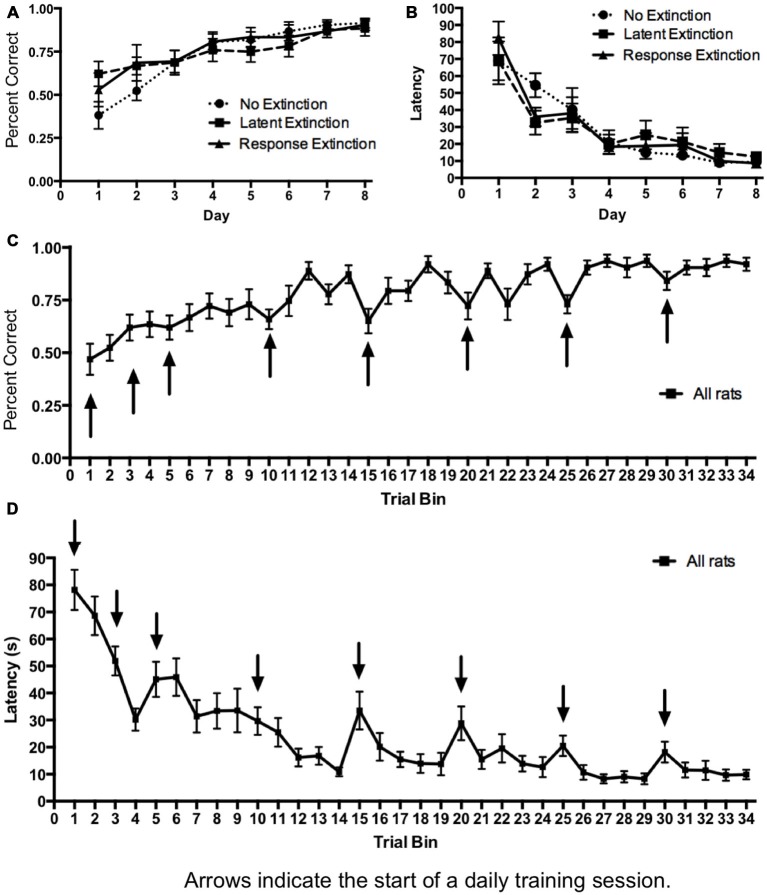
**Acquisition of hippocampus-dependent place learning in the plus-maze. (A,B)** The percentage of correct turns increased **(A)** and the latency to reach the correct food well decreased **(B)** over the course of training, with no differences between groups. **(C,D)** Subsequently, all groups were combined, and the trials of each day were averaged into trial bins (1 trial bin = 3 trials). Animals were more likely to make incorrect turns **(C)** and were slower **(D)** on the first few trials of a given training day vs. the last few trials of the previous day.

Initial acquisition of the place learning task is depicted in Figure 1. A two-way repeated measures 3 × 8 ANOVA (Group × Day) computed on percentage of correct turning responses over the course of training (Figure 1A) indicated a significant main effect of Day (*F*_(7,126)_ = 22.22, *p* < 0.001), but no effect of Group (*F*_(2,18)_ = 0.15, *p* = 0.860) and no Group × Day interaction (*F*_(14,126)_ = 1.51, *p* = 0.118). Likewise, a 3 × 8 ANOVA (Group × Day) computed on latency (Figure 1B) indicated a significant effect of Day (*F*_(7,126)_ = 52.41, *p* < 0.001), but no effect of Group (*F*_(2,18)_ = 0.00, *p* = 1.00) and no Group × Day interaction (*F*_(14,126)_ = 1.47, *p* = 0.131). Together, these results indicate that all groups acquired the task about equally over the course of training, and any subsequent differences between groups during extinction may not be readily attributed to differing rates of initial task acquisition.

#### Response Extinction

**Figure 2:**
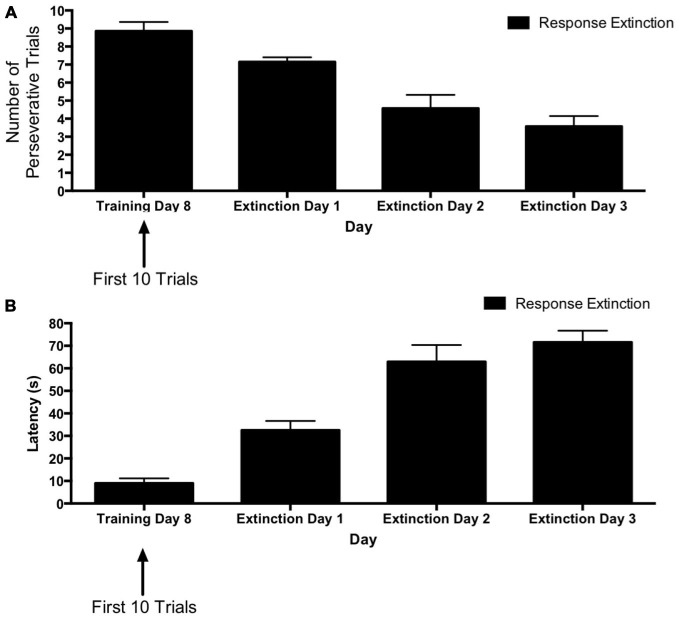
**Response extinction of hippocampus-dependent place learning. (A,B)** For animals in the response extinction group, the number of perseverative trials decreased **(A)** and latency increased **(B)** over the course of extinction training, indicating the effectiveness of response extinction.

Figure 2 depicts learning rates over the course of extinction training for animals in the “response extinction” group. Tests of within-subjects contrasts computed on number of perseverative trials (Figure 2A) revealed a significant linear effect of Day (*F*_(1,6)_ = 39.06, *p* = 0.001), indicating a decrease in number of perseverative trials during response extinction training. In addition, within-subjects contrasts computed on latency for extinction training days 1–3 (Figure 2B) also revealed a linear effect of Day (*F*_(1,6)_ = 113.56, *p* < 0.001), indicating that latency increased over the course of response extinction training.

#### Extinction Probes

**Figure 3:**
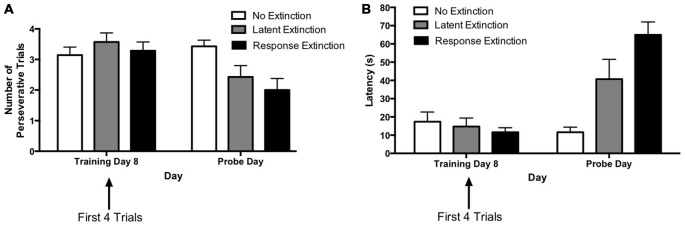
**Extinction probe trials in the hippocampus-dependent place learning task. (A)** There were no between-group differences in perseveration during the first few trials of the last training day (i.e., training day 8). Response and latent extinction groups, but not the “no extinction” group, displayed a decrease in number of perseverative trials from the last acquisition day to the probe day. On the probe day, the latent and response extinction groups displayed lower perseveration than the no extinction group, but the latent and response extinction groups did not differ from each other in perseveration. **(B)** There were no differences in latency between groups on the last training day. Response and latent extinction groups, but not the “no extinction” group, increased latency from the last acquisition day to the probe day. On the probe day, the latent and response extinction groups had higher latency than the no extinction group. Latency was also higher in the latent extinction group vs. the response extinction group on the probe day. Results indicate the effectiveness of latent and response extinction protocols in extinction of hippocampus-dependent place learning.

The results from the extinction probe trials are depicted in Figure 3. To assess the effectiveness of the different types of extinction training for each group, comparisons were made between the probe day and the last day of initial acquisition. The first four trials (vs. the last four trials) of the last acquisition day were selected for this comparison based on the observation that during initial acquisition, animals were typically slower and more likely to make errors for the first few trials of each training day vs. the final training trials of the previous day (see Figures 1C,D). Therefore, it was reasonable to expect that the extinction probe trials would also have higher latencies and more errors than the terminal trials of the last acquisition day, regardless of whether an extinction protocol was effective. Thus, for a more accurate measurement of the effectiveness of each extinction protocol, we compared the extinction probe trials with the *first* four trials of the final acquisition day.

A two-way repeated measures 3 × 2 ANOVA (Group × Day) was computed for number of perseverative trials on the last acquisition day (i.e., training day 8; first four trials) and the extinction probe day (Figure 3A). Results indicated no significant main effect of Group (*F*_(2,18)_ = 1.79, *p* = 0.195), but there was a significant effect of Day (*F*_(1,18)_ = 10.89, *p* = 0.004) and a significant Group × Day interaction (*F*_(2,18)_ = 5.37, *p* = 0.015). Multiple pairwise comparisons using Fisher’s LSD test indicated that there were no significant differences in number of perseverative trials between groups on the last acquisition day. This is consistent with data presented above indicating that the groups did not differ during initial task acquisition. For animals in the latent extinction group, Fisher’s LSD test indicated that there was a significant decrease in the number of perseverative trials from the last acquisition day (*M* = 3.57) to the probe day (*M* = 2.43), *p* = 0.007. In addition, the response extinction group showed a significant decrease in number of perseverative trials between the last acquisition day (*M* = 3.29) and the probe day (*M* = 2.00), *p* = 0.003. Animals given no extinction did not show a significant change in number of perseverative trials from the last acquisition day (*M* = 3.14) to the probe day (*M* = 3.43), *p* = 0.456. On the extinction probe day, Fisher’s LSD test indicated that the latent extinction group (*M* = 2.42) displayed a significantly lower number of perseverative trials than animals in the no extinction control group (*M* = 3.42), *p* = 0.026. Similarly, number of perseverative trials during probe day for the response extinction group (*M* = 2.00) was also significantly lower than perseverative trials for the no extinction group, *p* = 0.002. In contrast, perseverative trials for the latent extinction group and response extinction group did not differ on the probe day, *p* = 0.327.

A two-way repeated measures 3 × 2 ANOVA (Group × Day) was computed for latency on the last acquisition day (i.e., training day 8; first four trials) and the extinction probe day (Figure 3B). Results indicated a significant main effect of Group (*F*_(2,18)_ = 5.48, *p* = 0.014), a significant effect of Day (*F*_(1,18)_ = 36.84, *p* < 0.001), and a significant Group × Day interaction (*F*_(2,18)_ = 17.92, *p* < 0.001). Multiple pairwise comparisons using Fisher’s LSD test indicated that there were no significant differences in latency between groups on the last acquisition day. For animals given latent extinction, there was a significant increase in latency from the last acquisition day (*M* = 14.77) to the probe day (*M* = 40.71), *p* = 0.002. There was also a significant increase in latency between the last acquisition day (*M* = 11.61) and the probe day (*M* = 65.00) for animals given response extinction, *p* < 0.001. Animals given no extinction did not show a significant change in latency from the last acquisition day (*M* = 17.39) to the probe day (*M* = 11.61), *p* = 0.419. On the probe day, Fisher’s LSD test indicated that latency for the latent extinction group (*M* = 40.71) was significantly higher than latency in the no extinction control group (*M* = 11.61), *p* = 0.002. In addition, probe day latency for animals in the response extinction group (*M* = 65.00) was significantly higher than latency in the no extinction control group, *p* < 0.001. Latency in the response extinction group was also significantly higher than latency in the latent extinction group, *p* = 0.009.

Taken together, the results of Experiment 1 indicate that following acquisition in a place learning task animals given latent or response extinction displayed higher latency and lower perseveration during the extinction probe trials, relative to animals given no extinction. These results suggest that either a latent or response extinction protocol may be effective at extinguishing hippocampus-dependent place learning in the plus-maze.

### Experiment 2

#### Initial Acquisition

**Figure 4:**
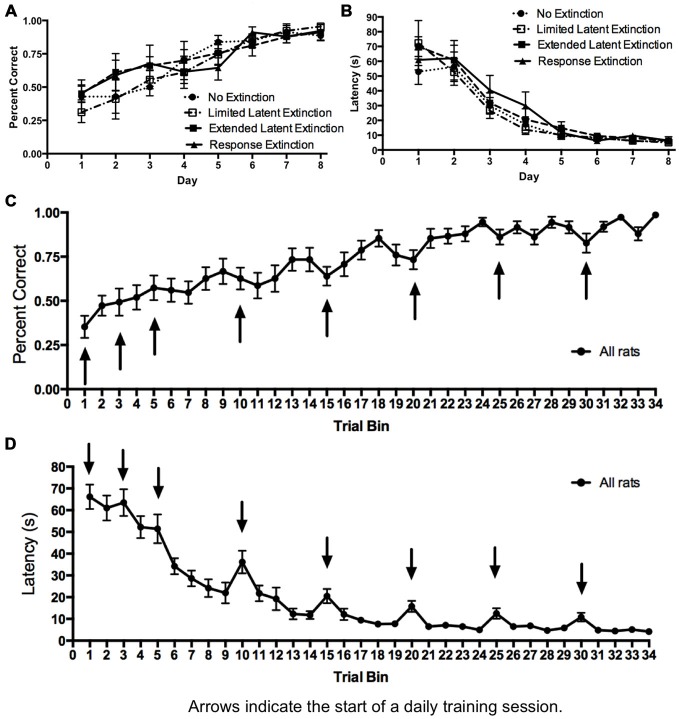
**Acquisition of DLS-dependent response learning in the plus-maze. (A,B)** The percentage of correct turns increased **(A)** and the latency to reach the correct food well decreased **(B)** over the course of training in the response learning task. There were no differences between groups, suggesting all groups acquired the task about equally. **(C,D)** All groups were combined, and the trials of each day were averaged into trial bins (1 trial bin = 3 trials). Animals were more likely to make incorrect turns **(C)** and were slower **(D)** on the first few trials of a given training day vs. the last few trials of the previous day.

Initial acquisition of the response learning task is depicted in Figure 4. A two-way repeated measures 4 × 8 ANOVA (Group × Day) computed on percentage of correct turning responses over the course of training (Figure 4A) indicated a significant main effect of Day (*F*_(7,147)_ = 23.74, *p* < 0.001), but no effect of Group (*F*_(3,21)_ = 0.224, *p* = 0.878) and no Group × Day interaction (*F*_(21,147)_ = 0.753, *p* = 0.771). Similarly, a two-way repeated measures 4 × 8 ANOVA (Group × Day) computed on latency (Figure 4B) also indicated a significant effect of Day (*F*_(7,147)_ = 95.52, *p* < 0.001), no effect of Group (*F*_(3,21)_ = 0.330, *p* = 0.800), and no Group × Day interaction (*F*_(21,147)_ = 0.88, *p* = 0.620). These results indicate that all groups acquired the task about equally. Therefore, any subsequent differences between groups during extinction may not be readily attributed to differing rates of initial task acquisition.

#### Response Extinction

**Figure 5:**
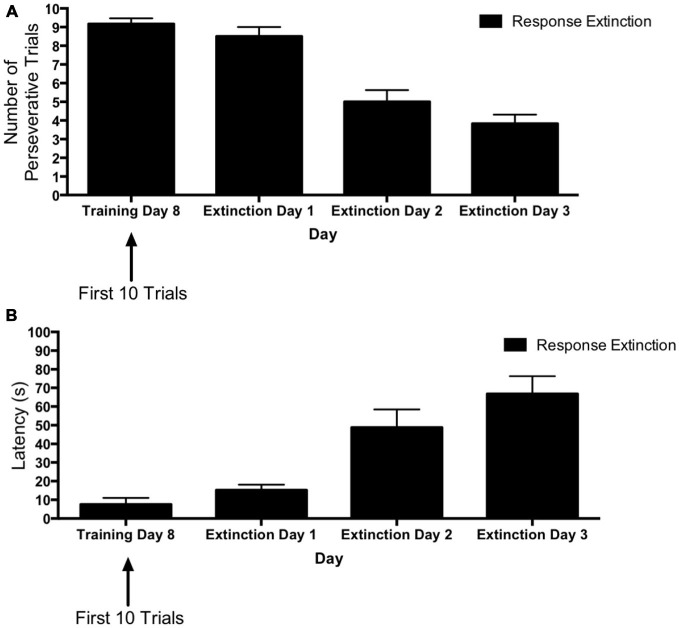
**Response extinction of DLS-dependent response learning. (A,B)** For animals in the response extinction group, the number of perseverative trials decreased **(A)** and latency increased **(B)** indicating the effectiveness of response extinction training.

Figure 5 depicts learning over the course of extinction training for animals in the “response extinction” group. Tests of within-subjects contrasts computed on number of perseverative trials (Figure 5A) for extinction days 1–3 revealed a significant linear effect of Day (*F*_(1,5)_ = 24.98, *p* = 0.004), indicating that the number of perseverative trials decreased over the course of response extinction training. In addition, within-subjects contrasts computed on latency (Figure 5B) also revealed a significant effect of Day (*F*_(1,5)_ = 23.90, *p* = 0.005), indicating that latency increased over the course of response extinction training.

#### Extinction Probes

**Figure 6:**
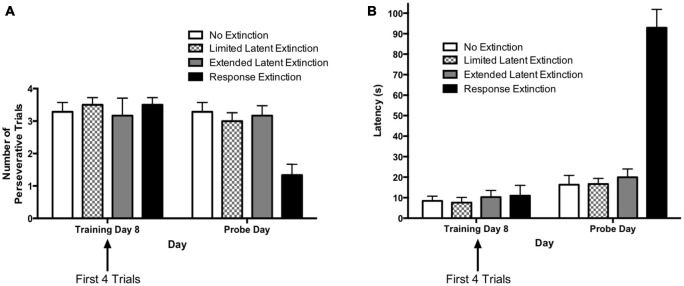
**Extinction probe trials in the DLS-dependent response learning task. (A)** There were no differences between groups in perseveration during the first few trials of the last acquisition day (i.e., training day 8). Only the response extinction group displayed a decrease in number of perseverative trials from the last acquisition day to the probe day. On the probe day, the response extinction group displayed lower perseveration than all other groups. The latent extinction groups (limited and extended) did not differ in perseveration from the no extinction control group on the probe day. **(B)** There were no between-group differences in latency on the last training day. All groups increased latency from the last acquisition day to the probe day. On the probe day, the response extinction group had higher latency than all other groups. Latency was not higher in the latent extinction groups (limited and extended), relative to the no extinction group. Results indicate that response extinction was effective and latent extinction was ineffective at extinguishing memory of DLS-dependent response learning.

The results from the extinction probe trials are depicted in Figure 6. The rationale for comparing extinction probe performance with the first four trials of the final training day was described in the results for Experiment 1 (see above). A two-way repeated measures 4 × 2 ANOVA (Group × Day) was computed for number of perseverative trials on the last acquisition day (i.e., training day 8; first four trials) and the extinction probe day (Figure 6A). Results indicated a significant main effect of Group (*F*_(3,21)_ = 3.73, *p* = 0.027), a significant effect of Day (*F*_(1,21)_ = 7.66, *p* = 0.012), and a significant Group × Day interaction (*F*_(3,21)_ = 4.48, *p* = 0.014). Multiple pairwise comparisons using Fisher’s LSD test indicated that there were no significant differences in number of perseverative trials between groups on the last acquisition day. This is consistent with data presented above indicating that the groups did not differ during initial task acquisition. For animals in the “response extinction” group, Fisher’s LSD test indicated that there was a significant decrease in the number of perseverative trials from the last acquisition day (*M* = 3.50) to the probe day (*M* = 1.33), *p* < 0.001. No other groups showed a significant change in number of perseverative trials between the last acquisition day and the probe day. On the extinction probe day, Fisher’s LSD test indicated that the response extinction group (*M* = 1.33) displayed a significantly lower number of perseverative trials than animals in the no extinction control group (*M* = 3.23), *p* < 0.001. Number of perseverative trials for the limited latent extinction group (*M* = 3.00) did not differ from the no extinction group, *p* = 0.642. In addition, perseverative trials for the extended latent extinction group (*M* = 3.17) did not differ from the no extinction group, *p* = 0.790. There was a significantly lower number of perseverative trials in the response extinction group vs. the limited latent extinction group, *p* < 0.001, and the extended latent extinction group, *p* < 0.001.

A two-way repeated measures 4 × 2 ANOVA (Group × Day) was computed for latency on the last acquisition day (i.e., training day 8; first four trials) and the extinction probe day (Figure 6B). Results indicated a significant main effect of Group (*F*_(3,21)_ = 22.00, *p* < 0.001), a significant effect of Day (*F*_(1,21)_ = 183.9, *p* < 0.001), and a significant Group × Day interaction (*F*_(3,21)_ = 81.57, *p* < 0.001). Multiple pairwise comparisons using Fisher’s LSD test indicated that there were no significant differences in latency between groups on the last acquisition day. Comparing the mean latencies between the last acquisition day and the probe day for each group indicated a significant increase in latency between the 2 days for all groups: no extinction (last acquisition day *M* = 8.46, probe day *M* = 16.32, *p* = 0.049), limited latent extinction (last acquisition day *M* = 7.58, probe day *M* = 16.67, *p* = 0.037), extended latent extinction (last acquisition day *M* = 10.29, probe day *M* = 19.96, *p* = 0.027), and response extinction (last acquisition day *M* = 11.00, probe day *M* = 92.92, *p* < 0.001). On the probe day, Fisher’s LSD test indicated that latency for the response extinction group (*M* = 92.92) was significantly higher than latency in the no extinction control group (*M* = 16.32), *p* < 0.001. Latency did not differ significantly between limited latent extinction (*M* = 16.67) and the no extinction control group, *p* = 0.957, and latency also did not differ between extended latent extinction (*M* = 19.96) and the no extinction control group, *p* = 0.567. Response extinction latency was significantly higher than latency in limited latent extinction, *p* < 0.001, and extended latent extinction groups, *p* < 0.001.

Taken together, the results of Experiment 2 indicate that following acquisition in the response learning task, animals given response extinction displayed higher latency and lower perseveration during the extinction probe trials, relative to animals given no extinction. In contrast, animals given limited or extended latent extinction protocols did not differ significantly in latency or perseveration from animals given no extinction. The results suggest that in contrast to typical response extinction, latent extinction protocols may not be effective at extinguishing memory in a DLS-dependent response learning task.

## Discussion

The present findings indicate a dissociation regarding the effectiveness of latent extinction across two learning and memory tasks. Latent extinction was effective at extinguishing memory in a hippocampus-dependent place learning task, but not in a DLS-dependent response learning task. In contrast, typical “response extinction” was effective in both place and response learning tasks.

In Experiment 1, following acquisition of the place learning task, animals given latent or response extinction displayed greater latency and fewer perseverative trials than animals given no extinction. Interestingly, animals given response extinction displayed higher latencies than animals given latent extinction, suggesting response extinction may have had greater efficacy than latent extinction in the place learning task. However, there was no difference in number of perseverative trials between latent and response extinction groups. It is possible that, relative to latent extinction, response extinction was more efficient at slowing the running approach response, but not necessarily more effective at extinguishing the location of food reward.

In Experiment 2, following acquisition of a response learning task, animals given response extinction displayed higher latencies and fewer perseverative trials than animals given no extinction, indicating the effectiveness of response extinction in this task. In contrast, animals given limited or extended latent extinction did not differ in latency or perseveration from animals given no extinction, suggesting that these latent extinction protocols were not effective at producing extinction in the response learning task. Even though latencies in the limited and extended latent extinction groups showed a slight increase from the last acquisition day to the probe day, a comparable increase was also observed for animals in the “no extinction” control group. Therefore, this increase in latency from the last acquisition day to the probe day may not be readily attributed to the latent extinction protocols. In addition, latent extinction and no extinction control groups did not show a decrease in number of perseverative trials across the 2 days.

**Figure 7:**
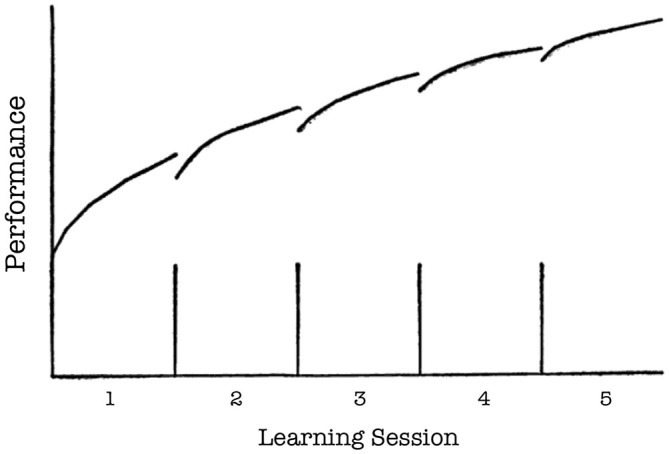
**Thorndike’s hypothetical model of memory decay and recovery.** The segmented linear curve indicates memory performance (*y* axis) over the course of five learning sessions (*x* axis). The vertical lines on the *x* axis indicate four periods of disuse (i.e., periods of time between sessions in which the memory is not retrieved). Performance decreases (i.e., decays) following each period of disuse. However, relearning during a subsequent session results in a stronger memory that is less sensitive to decay. Therefore, decay becomes progressively less pronounced following each subsequent period of disuse (From Thorndike, 1913, p. 283; axis labels added).

A finding secondary to the differential effects of the extinction protocols, but of considerable relevance to classical learning theories, pertains to the initial acquisition curves in the place and response learning tasks. During most days of initial acquisition, the first few trials were accompanied with greater latencies and more errors than the last few trials of the previous training day (see Figures 1C,D, 4C,D). However, this rise in latency and inaccuracy on the first few training trials of a given day became progressively less pronounced on subsequent training days. The present finding is consistent with early principles in learning theory pertaining to decay theory (e.g., Ebbinghaus, 1913; Thorndike, 1913). Thorndike (1913) proposed that following acquisition, a memory begins to fade as a function of its disuse over time (i.e., decay). However, some traces of the memory survive this decay, and thus relearning not only proves faster than initial learning, but also results in a stronger memory that is less sensitive to memory decay. Although the precise mechanisms of memory decay have been disputed (McGeoch, 1932), the general predictions of Thorndike’s model (see Figure 7) resemble the acquisition curves obtained in the present study. It is possible that some decay (or, more generally, forgetting) occurred in between daily training sessions, but that with each subsequent session of relearning the memory became more firmly ingrained and less sensitive to decay.

The principal finding that latent extinction was effective in the place learning task but not the response learning task may be related to differences between the memories acquired in each task. That is, latent extinction might only be effective when the to-be-extinguished memory contains certain critical features. The tasks selected for the present experiments depended on distinct neural systems, and solving each task hinged on different learning requirements. The hippocampus-dependent place learning task presumably required animals to encode the spatial location of the food reward to guide behavior to the correct arm, whereas the DLS-dependent response learning task only required that animals encode a left body-turn response at the maze choice point. Although animals being trained in the response learning task could also encode the spatial locations of the food reward, this information was not necessary for acquisition and ongoing performance in this task. In fact, extensive evidence indicates that spatial information might interfere with acquisition in the response learning task (for reviews, see Poldrack and Packard, 2003; Packard and Goodman, 2013).

Latent extinction in maze learning tasks might only be effective when the spatial location of the reinforcer is a critical part of the to-be-extinguished memory. Previous studies examining latent extinction have typically employed maze tasks, such as the straight alley maze, that could be solved adequately using either spatial or non-spatial learning strategies. In “dual-solution” tasks such as these, animals typically employ spatial learning strategies when the learning environment constitutes a heterogeneous visual surround, whereas animals employ response learning strategies when the task is conducted in a homogeneous visual surround (for reviews, see Restle, 1957; Packard and Goodman, 2013). Interestingly previous studies have indicated that latent extinction was only effective in heterogeneous visual surrounds conducive to allocentric spatial learning (e.g., Seward and Levy, 1949; Denny and Ratner, 1959; Dyal, 1962). Latent extinction was not effective in homogenous visual surrounds that prevented the use of allocentric spatial learning (e.g., Bugelski et al., 1952; Scharlock, 1954; Denny and Ratner, 1959). These previous findings are consistent with the suggestion that in maze learning tasks, latent extinction might be selectively effective at extinguishing allocentric spatial memory.

The finding that latent extinction might only be successful at extinguishing certain types of memory could be attributed to the distinct learning mechanisms through which latent extinction operates. Unlike response extinction, latent extinction does not conform to classical models of extinction that suggest the animal must make the previously acquired response for extinction to occur (e.g., Hull, 1943, 1952). Proponents of the Hullian S-R view of learning have suggested that latent extinction, although it may not be readily explained by Hull’s traditional response-inhibition theory of extinction, could still be accounted for through a Hullian fractional anticipatory response mechanism (Hull, 1931; Spence, 1951). According to this view (Moltz, 1957), an unobservable component of the consumatory goal response is elicited by cues throughout the maze during initial acquisition of the task, and this partially guides behavior to the correct goal location. When an animal is confined to the goal box during latent extinction, this fractional goal response is elicited and over time, becomes extinguished to the goal box cues. To the extent that the goal box cues might resemble earlier sections of the maze, extinction of the fractional goal response will generalize to other parts of the maze, resulting in increased latency and incorrect turns during extinction probe trials. Several cogent arguments have been raised indicating the inadequacy of this potential S-R mechanism in explaining latent extinction (Gleitman et al., 1954; Treisman, 1960). In addition, this putative mechanism is not supported by the present findings. If latent extinction were to operate by extinguishing a fractional response in the goal box that generalizes to other parts of the maze, then it would be reasonable to predict that latent extinction would be effective across both place and response learning tasks, which presently was not observed.

Previous evidence from our laboratory suggests that latent extinction may involve spatial memory mechanisms (Gabriele and Packard, 2006). Temporary inactivation of the dorsal hippocampus with bupivacaine blocks the effectiveness of latent extinction in the straight alley maze (Gabriele and Packard, 2006). Considering that a principal function of the hippocampus involves spatial memory formation (O’Keefe and Nadel, 1978; Morris et al., 1982), it is possible that hippocampal inactivation blocked latent extinction by disrupting hippocampus-dependent spatial memory processing. That latent extinction might depend in part on spatial memory processing is largely consistent with previous behavioral evidence. As mentioned previously, latent extinction is selectively effective in heterogeneous visual environments conducive to spatial memory formation, but not homogenous visual environments that prevent spatial memory formation (Seward and Levy, 1949; Bugelski et al., 1952; Scharlock, 1954; Denny and Ratner, 1959; Dyal, 1962).

Latent extinction may involve spatial memory processing insofar as confining an animal to a previously rewarded spatial location without food (i.e., latent extinction) might allow the animal to acquire a new memory in which the spatial location becomes associated with absence of food. Thus, for latent extinction to be successful, a rat must be confined to the previously rewarded spatial location. Confining a rat to an empty goal box located in a different room (Iwahara et al., 1953) or a different spatial location in the same room (Clifford, 1964) does not produce extinction. This proposed mechanism for latent extinction is consistent with its dependance on hippocampal function, i.e., in addition to acquiring information about food rewarded locations, the hippocampus is similarly involved in linking spatial locations with the *absence* of food reward (Gaskin and White, 2006).

This putative spatial mechanism could also explain why latent extinction was effective in the place learning task, but not the response learning task. In the place learning task, memory performance was presumably guided by a learned association in which a spatial location had been associated with the food reward. Thus, if the same spatial location were subsequently associated with the absence of food reward, which putatively occurs during latent extinction, we should expect memory performance in the place learning task to decline. In contrast, memory performance in the response learning task was presumably not guided by the spatial locations of the food reward, and therefore associating spatial locations with the absence of food reward should not affect later retrieval of the previously acquired response.

Given the effectiveness of typical response extinction across both place and response learning tasks, it is tempting to speculate that response extinction might depend on a distinct learning mechanism. Previous evidence from our laboratory indicates that in contrast to latent extinction, the effectiveness of response extinction in the straight alley maze is not impaired following hippocampal inactivation (Gabriele and Packard, 2006). Rather, response extinction in the straight alley maze is attenuated following lesion or temporary inactivation of the DLS (Dunnett and Iversen, 1981; Thullier et al., 1996; Gabriele, 2008). Considering that the DLS is a chief neural substrate implicated in S-R learning and memory processes (Packard and Knowlton, 2002), one possibility is that during response extinction the DLS forms S-R associations between visual cues in the learning situation (i.e., the stimuli) and the inhibition of a behavior (i.e., the response). Several investigators have proposed similar S-R mechanisms to account for extinction across maze learning, operant lever pressing, and Pavlovian conditioning paradigms (Guthrie, 1935; Hull, 1943; Rescorla, 1993; Delamater, 2004). Importantly, the learned inhibition of behavior during response extinction could potentially explain the effectiveness of this protocol in both place learning and response learning tasks.

Aside from the direct involvement of multiple memory systems, another potential mechanism underlying the selective effectiveness of latent extinction pertains to the immediate differences between the two tasks. Although the place and response learning tasks were identical in terms of their motivational, sensory, and motoric requirements, it was necessary that the tasks differed slightly in some respects so that each task invoked a different memory system. We cannot rule out the possibility that slight differences between the two tasks (e.g., in the place learning task, animals received food in one location; in the response learning task, animals received food in two locations) may have partially influenced the effectiveness of latent extinction.

In sum, the present findings indicate that whereas response extinction successfully extinguished memory in hippocampus-dependent place learning and DLS-dependent response learning tasks, latent extinction was selectively effective in the place learning task and not the response learning task. The suggestion that the principal learning mechanisms underlying latent extinction involve an acquired association between the spatial location and the absence of food reward may provide an explanation for the selective effectiveness of latent extinction across these learning tasks. Future studies utilizing a wider variety of spatial and non-spatial memory tasks are required to further examine this hypothesis.

## Author Contributions

JG contributed ideas, manuscript writing, and conducted research. MGP contributed ideas and manuscript writing.

## Conflict of Interest Statement

The authors declare that the research was conducted in the absence of any commercial or financial relationships that could be construed as a potential conflict of interest.
